# Expression of IL-33 Receptor Is Significantly Up-Regulated in B Cells During Pregnancy and in the Acute Phase of Preterm Birth in Mice

**DOI:** 10.3389/fimmu.2020.00446

**Published:** 2020-03-27

**Authors:** Natalin Valeff, Lorena Juriol, Florencia Quadrana, Damián Oscar Muzzio, Marek Zygmunt, Maria Florencia Quiroga, María Silvia Ventimiglia, Federico Jensen

**Affiliations:** ^1^Laboratory for Immunology of Pregnancy, Center for Pharmacological and Botanical Studies (CEFYBO-CONICET-UBA), Buenos Aires, Argentina; ^2^Research Laboratory, Department of Obstetrics and Gynecology, University of Greifswald, Greifswald, Germany; ^3^Instituto de Investigaciones Biomédicas en Retrovirus y Sida (INBIRS), Facultad de Medicina, Universidad de Buenos Aires, Consejo Nacional de Investigaciones Científicas y Técnicas (CONICET), Buenos Aires, Argentina; ^4^Institute of Health Sciences, National University Arturo Jauretche, Buenos Aires, Argentina

**Keywords:** B cells, pregnancy, preterm birth, IL-33, ST2, tolerance

## Abstract

Interleukin-33 (IL-33) is a mucosal alarmin belonging to the IL-1 cytokine family and is now recognized to have a key role in innate and adaptive immunity, contributing to tissue homeostasis and response to environmental stresses. In addition, IL-33 has also been shown to work as a positive regulator that initiates and maintains a Th2 immune response. In the context of pregnancy, it has been recently demonstrated that upon certain stress conditions, such as an infection induced inflammation, IL-33 is released from the uterine mucosa and triggers decidual B cells to produce anti-inflammatory molecules, which in turn restore immune homeostasis and prevents the development of preterm birth. In this study we therefore performed a detailed characterization of IL-33 receptor (*Il1rl1* or ST2) expression in B cells during normal pregnancy, as well as in a mouse model of preterm birth. We observed that splenic B cells significantly up-regulate the expression of *Il1rl1* during pregnancy and identified the B1 B cell population as the main ST2-expressing B cell subset. A further kinetic analysis showed that percentages of ST2-expressing B1 B cells are significantly augmented on days 12 and 14 of pregnancy, both in the spleen and peritoneal cavity of pregnant mice, and then drop toward the end of pregnancy to the levels observed in non-pregnant animals. Furthermore, using a mouse model of LPS-induced preterm birth, we demonstrated that not only are the percentages of ST2-expressing B1 B cells significantly enlarged in the spleen during the acute phase of preterm birth, but decidual B cells also significantly up-regulate ST2 expression as compared to term-pregnant mice. Overall, our results suggest a functional role of ST2 expression in B cells during pregnancy and reinforce the importance of the IL-33/ST2 axis in B cells as a critical mechanism to control inflammation-induced preterm birth.

## Introduction

Successful pregnancies in mammals rely on the capacity of the maternal immune system to finely regulate an immune balance between immune tolerance, to avoid rejection of the semi-allogeneic fetus, and immune activation, to protect the mother against infection. Different immune mechanisms, including innate as well as adaptive immunity, have been implicated in this phenomenon; failures in the acquisition or disruption of this intricate immune balance might lead to pregnancy complications (1–11).

Our laboratory and others have extensively demonstrated that the B cell compartment undergoes significant adaptations during pregnancy, apparently to support the acceptance of the semi-allogeneic fetus (12–19) and to avoid the occurrence of immune mediated pregnancy disorders, like preterm birth (PTB) among others (15, 19–30). Moreover, it has recently been demonstrated that decidual B cells, through a mechanism involving IL-33, can control inflammation induced by bacterial components and significantly reduce the occurrence of PTB (31).

IL-33 is a member of the IL-1 family of cytokines (32–34), produced and released by damaged or necrotic endothelial and epithelial cells. IL-33 functions as an alarm signal (alarmin) released upon cell injury or tissue damage to alert immune cells expressing the IL-33 receptor (ST2) (35–37). Indeed, the main targets of IL-33 are tissue–resident immune cells, including B cells (38).

In order to get a deeper understanding of the immune mechanisms involved in normal pregnancy and in PTB, in the present study, we performed for the first time a detailed immunophenotyping study of the dynamics of ST2 expression in different B cell subsets during normal pregnancy, as well as in a mouse model of LPS-induced PTB.

## Materials and Methods

### Animals

Six to eight week-old C57B6/J females and male mice were purchased from the Central Animal Facility of the Faculty of Natural Sciences, Buenos Aires University. Mice were kept in our animal facility under optimal conditions in a 12L:12D cycle and fed *ad libitum*. Animal experiments were carried out according to institutional guidelines after approval by the Institutional Commission for the Care and Use of Laboratory Animals (CICUAL 2248).

Two C57BL6/J females were placed with a BALB/c male in the same cage and checked daily for vaginal plugs. The day vaginal plug was detected was considered day 0 of pregnancy. Pregnant females were separated from the males and euthanized at day 12 post-plug (dpp), 14 dpp, or 16 dpp. As control, non-pregnant (NP) age-matched virgin C57BL6/J females were included.

### Mouse Model of Preterm Birth

BALB/c pregnant C57B6/J females were challenged on day 16 of gestation with an IP injection of LPS (10 μg/mice), a dose that induces 100% of preterm birth in mice (unpublished data from our laboratory). PTB was defined as the delivery of a pup occurring between 24 and 48 h post LPS injection. To analyze ST2 expression in splenic and decidual B cells during the acute phase of PTB, animals were sacrificed 5 h after LPS challenge.

### Antibodies and Reagents

The following anti-mouse fluorescently labeled antibodies were used:

CD45 (clone 30-F11) B220 (clone RA3-6B2), CD19 (clone 6D5), CD23 (clone B3B4), CD21 (clone 7E9), CD5 (clone 53-7.3), and ST2 (clone DIH9). All antibodies were purchased from Biolegend USA.

### Cell Preparation and Flow Cytometry

For flow cytometry analysis, single-cell suspensions were obtained from different tissues following previously described procedures (17, 18, 39). Briefly, spleens were crushed in a 100-μm cell strainer to obtain a single cell suspension and red blood cells were lysed in lysis buffer 1X (NH_4_Cl 150 mM, KHCO_3_ 10 mM, and EDTA 1 mM) for 5 min. Peritoneal cell suspensions were obtained by peritoneal washouts with 10 ml PBS+BSA 3% (w/v). Cell isolation from decidual tissue was performed using a commercial enzymatic solution (StemPro Accutase, Gibco). Briefly, decidual tissues were dissociated in 500 μl of enzymatic solution with sterile scissors for 2 min on ice until solution was finely minced. Homogenized tissues were next incubated in enzymatic solution for 35 min at 37°C with gentle orbital agitation (80 rpm). Dissociated tissues were filtered through a cell strainer (100 μm), washed twice with phosphate-buffered saline (PBS, 1X), and centrifuged at 1,250 g for 10 min at 4°C. Cell pellets were resuspended in 1 ml of RPMI, loaded into a 15-ml falcon tube containing 500 μl of fetal bovine serum (FBS) and centrifuged at 1,100 g for 10 min at room temperature. After discarding cellular debris retained at the PBS/FBS interface, viable cells at the bottom of the tube were resuspended in PBS 1X.

After washing, cell suspensions were counted using a Neubauer chamber, and 1 × 10^6^ cells were stained for 30 min at 4 °C with specific labeled antibodies. Fluorescence minus one (FMO) was used as control. Data were acquired on FACS Canto II and BD Accuri C6 Plus (BD Biosciences) and analyzed by using FlowJo v10 software.

### RNA Isolation, cDNA Synthesis, and qPCR

Magnetically isolated splenic B cells (Pan B Cell Isolation Kit, Miltenyi Biotec) from pregnant (day 14 of gestation) and non-pregnant females were treated with TriFast peqlab. RNA isolation and first-strand cDNA synthesis (SuperScript™ III First-Strand Synthesis System, Invitrogen) were performed according to manufacturer's instruction. Primer pairs were designed using Primer Express® Primer Design Software v3.0 (Applied Biosystems) and chosen to span an exon–exon junction to avoid unwanted genomic DNA amplification. Primer sequences were as follows:

il1rl1 Fw: CCAGTAAGTGAGACAGCAGCATTT; il1rl1 Rw: CTGTAGATACCCAGATGAAGGGCT; β-actin Fw: TGGAATCCTGTGGCATCCATGAAAC; Rw: TAAAACGCAGCTCAGTAACAGTCC. All primers were purchased from Invitrogen (Carlsbad, CA, USA). CFX96 Real-Time PCR Detection Systems was used to perform semiquantitative qPCR (Power SYBR Green Master Mix, Applied Biosystems) with β-actin as housekeeping gene. Samples were amplified in duplicate and non-template samples were used as controls. Results were analyzed by BioRad CFX Manager 2.0. Relative expression was calculated using the following formula:

Relative expression = 2^−ΔCT^; where ΔCT = CT gen – CT β-actin.

### Statistical Analysis

Data were analyzed with PRISM software (ver. 5.01; GraphPad). The Shapiro–Wilk test was used to test for normality of the data. ANOVA followed by Tukey multiple post *t*-test (normally distributed data) or Kruskal–Wallis test followed by Dunn's multiple comparisons test (non-normally distributed data) was applied to evaluate differences between multiple groups. Two tailed *t*-test (normally distributed data) or Mann–Whitney *U*-test (non-normally distributed data) were applied to compare two groups. A *p* < 0.05 was considered statistically significant for all tests.

## Results

### *Ilrl1* Gene Expression Is Significantly Up-Regulated in Splenic B Cells During Pregnancy

**Figure 1:**
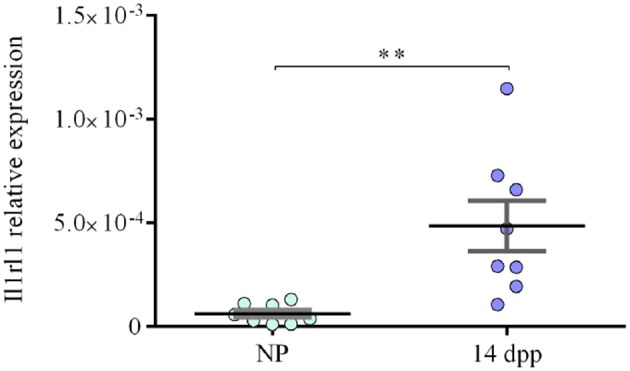
Expression levels of *Il1rl1* in pure isolated B cells from pregnant and non-pregnant mice. qPCR analysis showing significantly higher relative expression levels of *Il1rl1* normalized to β-actin, in splenic B cells isolated from pregnant mice on day 14 of pregnancy (P, *n* = 8) as compared to B cells isolated from non-pregnant (NP, *n* = 8) control females (6 × 10^−5^ ± 1 × 10^−5^ vs. 4 × 10^−4^ ± 1 × 10^−4^). Data is shown as mean ± SEM. **p < 0.01 as analyzed by unpaired *t-*test.

Unpublished data from a genome-wide transcriptomic profiling performed in our laboratory showed that levels of interleukin 1 receptor-like 1 mRNA (*Ilrl1*) codifying for IL-33 protein membrane receptor also known as ST2 or IL-33R were significantly up-regulated in splenic B cells isolated from pregnant mice on day 14 of pregnancy as compared to B cells isolated from the spleen of control mice (NP) (Unpublished data). Based on this data we began this work by analyzing the expression levels of *Ilrl1* in pure isolated splenic B cells from NP and pregnant (P) mice on day 14 of pregnancy by qPCR. As shown in Figure 1, expression levels of *Ilrl1* were significantly higher in isolated splenic B cells from pregnant mice on day 14 of pregnancy, as compared to B cells isolated from non-pregnant (NP) controls animals (Figure 1).

### Percentages of ST2-Expressing B Cells Are Augmented in the Spleen of Pregnant Mice

**Figure 2:**
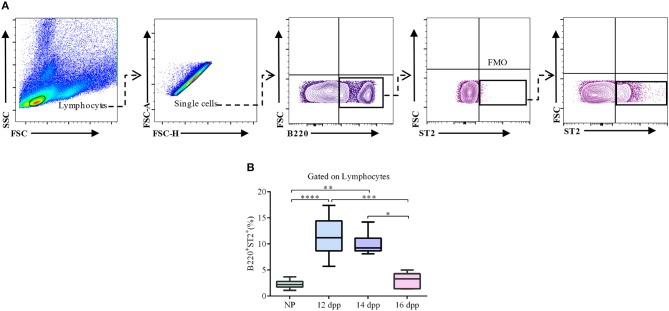
Kinetic analysis of ST2 expression in splenic B cells throughout pregnancy. **(A)** Representative pseudocolor and contour plots showing gating strategy used to quantify percentages of ST2-expressing B cells in the spleen of pregnant and non-pregnant females. Lymphocytes were gated by FSC vs. SCC, doublets were eliminated, and B220 was used to define total B cell population in the spleen. Fluorescence minus one (FMO) was used as control. **(B)** Box and whisker plots showing percentages of ST2-expressing total B cells in the spleen of pregnant mice on day 12 (12 dpp; *n* = 9), 14 (14 dpp; *n* = 11), and 16 (16 dpp; *n* = 7) of pregnancy as well as in non-pregnant control females (NP, *n* = 11). Data are expressed as Box and Whisker plots showing median. *p < 0.05; **p < 0.01; ***p < 0. 001; and ****p < 0.0001 as analyzed by Kruskal–Wallis test followed by Dunn's multiple comparisons test.

Once we confirmed that *Ilrl1* is up-regulated in splenic B cells toward mid-pregnancy, we next performed a kinetic analysis of ST2 expression, at protein levels, in splenic B cells through gestation. As shown in Figure 2, percentages of ST2-expressing B220^+^ B cells in the spleen of pregnant mice were significantly increased on day 12 of pregnancy and remained significantly high on day 14 as compared to NP control females (Figure 2). However, toward end of pregnancy (16 dpp), percentages of ST2-expressing B220^+^ B cells dropped to the levels observed in NP control mice (Figure 2).

### ST2 Is Predominantly Expressed in Splenic B1 B Cells During Pregnancy

**Figure 3:**
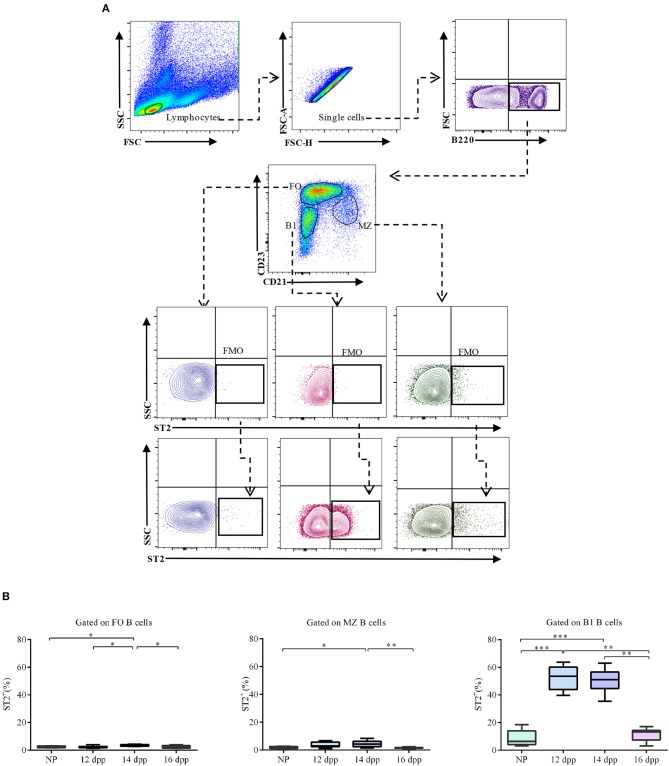
Percentages of ST2-expressing follicular, marginal zone, and B1 B cells in the spleen of pregnant and non-pregnant mice. **(A)** Representative pseudocolor and contour plots showing gating strategy used to quantify percentages of ST2-expressing follicular (FO), marginal zone (MZ), and B1 B cells in the spleen of pregnant and non-pregnant females. Lymphocytes were gated by FSC vs. SCC, doublets were eliminated, and B220 was used to define total B cells. CD23 and CD21 markers were used to identify FO (B220^+^CD23^hi^CD21^low^), MZ (B220^+^CD23^low^CD21^hi^), and B1 (B220^low^CD23^−^CD21^−^) B cells. Fluorescence minus one (FMO) was used as control. **(B)** Box and Whisker plots showing percentages of ST2-expressing FO, MZ and B1 B cells at day 12 (12 dpp; *n* = 9, *n* = 9, and *n* = 9, respectively), 14 (14dpp; *n* = 11, *n* = 11, and *n* = 11, respectively) and 16 (16 dpp; *n* = 7, *n* = 7, and *n* = 7, respectively) of pregnancy as well as in non-pregnant females (NP; *n* = 11, *n* = 11, and *n* = 11, respectively). Data are expressed as box and whisker plots showing median. *p < 0.05; **p < 0.01; ***p < 0.0001 as analyzed by ANOVA followed by Tukey's multiple comparisons test.

Next, we were interested in knowing if the increase observed in the percentages of splenic ST2-expressing B cells during pregnancy was homogeneously distributed through all B cell subsets or associated with a specific B cell sub-population. To address this, we used a well-defined splenic gating strategy based on the expression of B cell surface markers B220, CD21 and CD23 (40). By doing so, we analyzed the expression of ST2 in B1 (B220^low^CD23^−^*CD*21^−^), as well as follicular (FO: B220^+^CD23^hi^*CD*21^low^) and marginal zone (MZ: B220^+^*CD*23^low^*CD*21^hi^) B2 B cells (Figure 3). We found that percentages of ST2-expressing FO as well as MZ B cells were very low (Figure 3), and no differences were observed when comparing NP with pregnant mice on day 12 of pregnancy (2.4 ± 0.3 vs. 2.3 ± 0.2% and 3.7 ± 0.6 vs. 1.9 ± 0.3%, respectively). However, on day 14 of pregnancy, a modest but significant increase of ST2 expressing FO and MZ B cells was observed when compared to non-pregnant control mice (3.5 ± 0.2 vs. 2.3 ± 0.2% and 4.4 ± 0.7 vs. 1.9 ± 0.3%, respectively; Figure 3). At day 16 of pregnancy, levels of ST2- expressing FO and MZ B cells were similar to those observed in NP-control mice (2.3 ± 0.4 vs. 2.3 ± 0.2% and 1.7 ± 0.2 vs. 1.9 ± 0.3%, respectively). Interestingly, we detected a considerable increase in the percentages of ST2 expressing B1 B cells in the spleen of pregnant mice on day 12 and 14 of pregnancy as compared to NP mice (51.9 ± 2.9% and 51.1 ± 2.3 vs. 8.4 ± 2.1%, respectively), which was mirrored by a significant increase in ST2 mean fluorescence intensity (MFI; Supplementary Figure 1). At day 16 of pregnancy, percentages of ST2 expressing B1 B cells as well as ST2-MFI were similar to NP control mice (Figure 3 and Supplementary Figure 1, respectively).

### Levels of ST2-Expressing B1 B Cells Are Augmented in the Peritoneal Cavity During Pregnancy

**Figure 4:**
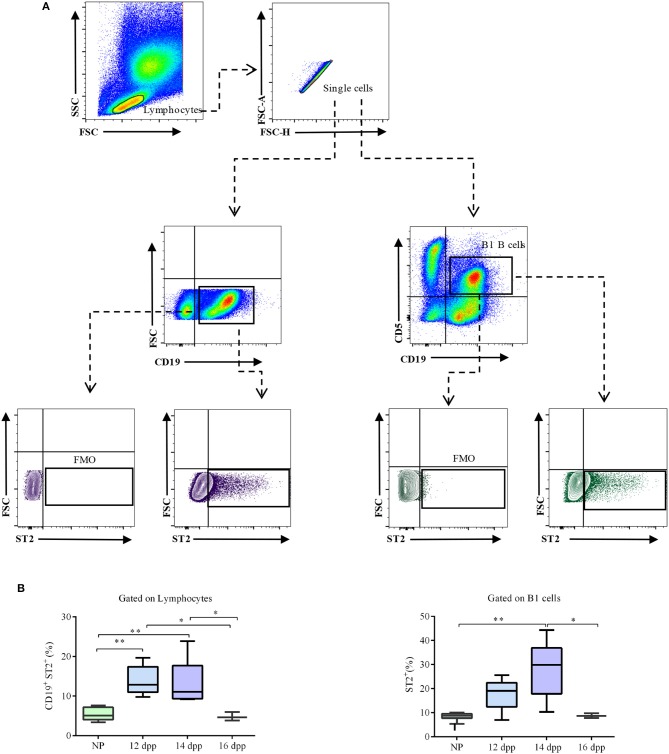
Percentages of ST2-expressing total and B1 B cells in the peritoneal cavity of pregnant and non-pregnant mice. **(A)** Representative pseudocolor and contour plots showing gating strategy used to quantify percentages of ST2-expressing total CD19^+^ B cells as well as CD19^+^CD5^+^ B1 B cells in the peritoneal cavity of pregnant and non-pregnant mice. Lymphocytes were gated by FSC vs. SCC, doublets were eliminated, and CD19 alone or in combination with and CD5 were used to define total as well as B1 B cells, respectively. Fluorescence minus one (FMO) was used as control. **(B)** Box and whisker plots showing percentages of ST2-expressing CD19^+^ B cells in peritoneal cavity of non-pregnant (NP; *n* = 9) and pregnant mice at day 12 (12 dpp; *n* = 5), 14 (14 dpp; *n* = 9), and 16 (16 dpp; *n* = 3) of pregnancy as well as percentages of ST2-expressing CD19^+^CD5^+^ B1 B cells in non-pregnant (NP; *n* = 9) and in pregnant mice at day 12 (12 dpp; *n* = 5), 14 (14 dpp; *n* = 9), and 16 (16 dpp; *n* = 3) of pregnancy. Data are expressed as box and whisker plots showing median. *p < 0.05; **p < 0.01 as analyzed by ANOVA followed by Tukey's multiple comparisons test.

As peritoneal cavity is the natural reservoir of the B1 B cells, we next analyzed the dynamic of ST2 expression in peritoneal cavity B cells during pregnancy (Figure 4A). Similarly to what was observed in the spleen, percentages of ST2-expressing CD19^+^ total B cells were significantly higher in the peritoneal cavity of pregnant mice on days 12 and 14 of pregnancy as compared to non-pregnant virgin females (14 ± 1.7% and 13.5 ± 1.8 vs. 5.5 ± 0.5%, respectively; Figure 4B). At day 16 of pregnancy, percentages of ST2-expressing B cells were similar to those observed in NP control mice (Figure 4B). Similarly, percentages of ST2-expressing CD19^+^CD5^+^ B1 B cells were increased, although not significantly, on day 12 of pregnancy (17.8 ± 3 vs. 8.5 ± 0.5%; Figure 4B) and reached statistical significance on day 14 of pregnancy, as compared to NP control mice (28.6 ± 3.7^**^ vs. 8.5 ± 0.5%; Figure 4B). At day 16 of pregnancy, percentages of ST2-expressing CD19^+^CD5^+^ B1 B cells were similar to those in NP control mice (8.5 ± 0.5 vs. 8.5 ± 0.5%; Figure 4B).

### Percentages of Splenic ST2-Expressing B1 B Cells Are Augmented in the Acute Phase of LPS-Induced PTB

**Figure 5:**
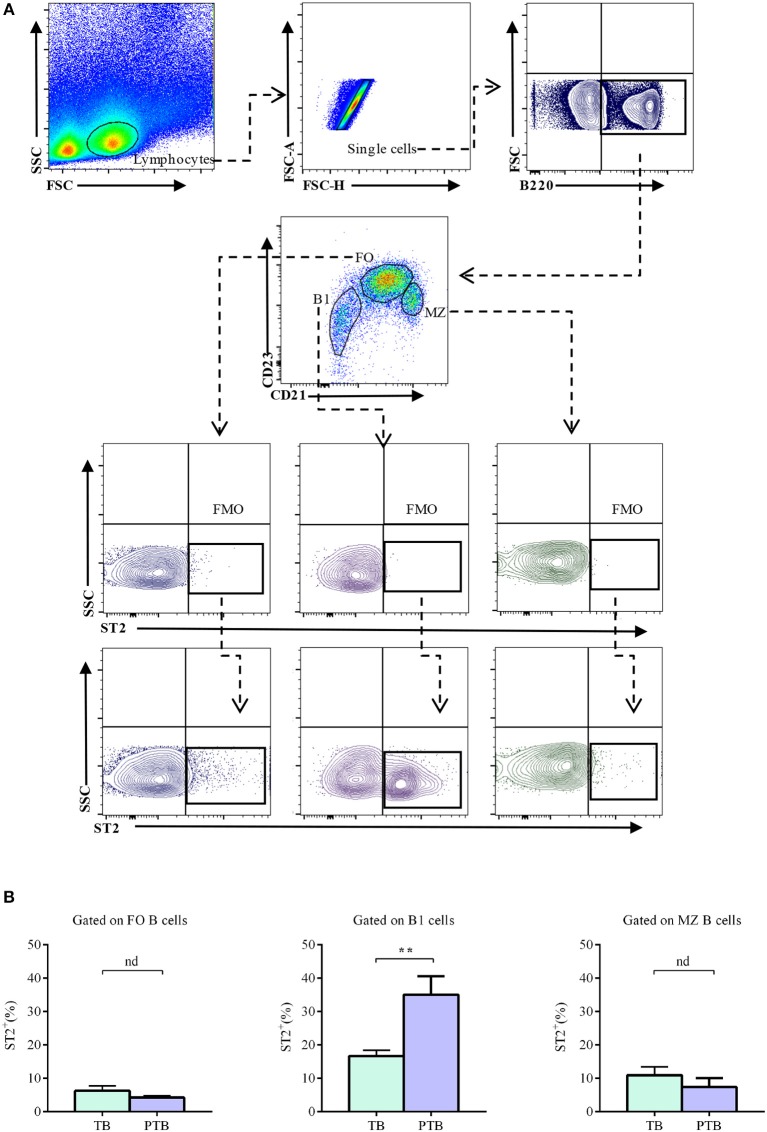
Percentages of ST2-expressing follicular, marginal zone, and B1 B cells in the spleen of pregnant mice during the acute phase of LPS-induced preterm birth. **(A)** Representative pseudocolor and contour plots showing gating strategy used to quantify percentages of ST2-expressing follicular (FO), marginal zone (MZ), and B1 B cells in the acute phase of LPS-induced preterm birth and in term-pregnant control mice. Lymphocytes were gated by FSC vs. SCC, doublets were eliminated and B220 was used to define total B cells. CD23 and CD21 markers were used to identify FO (B220^+^CD23^hi^CD21^low^), MZ (B220^+^CD23^low^CD21^hi^) and B1 (B220^low^CD23^−^CD21^−^) B cells. Fluorescence minus one (FMO) was used as control. **(B)** Bar graphs showing percentages of ST2-expressing FO, MZ, and B1 B cells in preterm birth (PT; *n* = 6) and term birth control mice (TB; *n* = 6). Data are expressed as mean ± SEM. **p < 0.01 as analyzed by two-tailed unpaired *t-*test.

It has recently been demonstrated that B cells play a critical role in protecting mice against inflammation-induced PTB through a mechanism involving mucosal alarmin IL-33 (31). Thus, we next analyzed the expression of ST2 in splenic B cells during the acute phase of LPS-induced PTB (5 h after LPS challenge). As shown in Figure 5, percentages of ST2-expressing follicular as well as marginal zone B cells in the spleen were similar in LPS-challenged mice (5 h after injection) compared to PBS-treated control mice (4.2 ± 0.5 vs. 6.3 ± 0.5 and 4.7 ± 2.6 vs. 10.9 ± 2.5; Figure 5). Remarkably, a significant expansion in the percentages of ST2-expressing B1 B cells was observed in the spleen of LPS-induced PTB mice, as compared to control mice (35 ± 5.6 vs. 16.7 ± 1.7; Figure 5).

### Decidual B Cells Up-Regulate ST2 Expression During the Acute Phase of PTB

It was recently demonstrated that, upon uterine stress provoked by LPS-induced inflammation, decidual cells respond producing IL-33, which in turn induces the production of anti-inflammatory molecules by decidual B cells, thus preventing or limiting induction of PTB. Hence, we next analyzed the expression of ST2 in decidual B cells during the acute phase of LPS-induced PTB.

**Figure 6:**
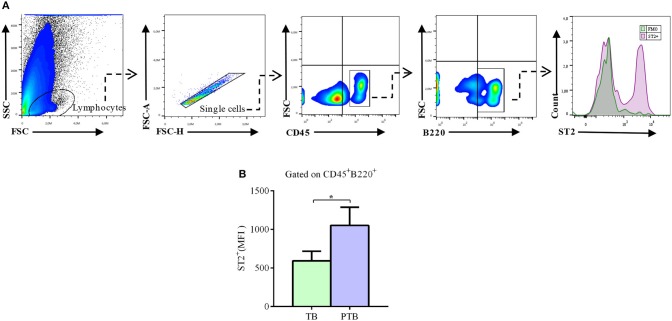
Expression levels of ST2 in decidual B cells during the acute phase of LPS-induced preterm birth. **(A)** Representative pseudocolor plots and histogram showing gating strategy used to quantify the expression of ST2 in decidual B cells during the acute phase of LPS-induced preterm birth (PTB) and in term birth mice (TB). Lymphocytes were gated by FSC vs. SCC and doublets were eliminated. After gating in total CD45^+^ leukocytes, B220 was used to identify total decidual B cells. Fluorescence minus one (FMO) was used as control. **(B)** Bar graph shows median of fluorescence intensity (MFI) for ST2 in decidual B cells from TB (*n* = 6) and PTB mice (*n* = 6). Data is expressed as mean ± SEM. *p < 0.05 as analyzed by Wilcoxon test.

We observed that 5 h after LPS injection, decidual CD45^+^B220^+^ B cells express significantly higher levels of ST2, as compared to PBS-treated pregnant mice undergoing term pregnancies (Figure 6).

## Discussion

We demonstrated in this work that splenic B cells significantly up-regulate IL-33 receptor at the RNA (*Ilrl1*) as well as at the protein (ST2) levels during physiological pregnancies in mice and identified the B1 B cell subset as the main B cell population expressing ST2. This is in agreement with previous publications indicating that ST2, in mice, is predominantly expressed in B1, while almost absent in B2 B cells (38). Furthermore, we showed that splenic as well as peritoneal cavity ST2-expressing B1 B cells followed a unique kinetic during pregnancy, being significantly high on days 12 and 14 of pregnancy and then dropping toward end of pregnancy (16 dpp) to levels observed in non-pregnant control mice. This special pattern of expression suggests that ST2-expresssing B1 B cells might play a differential role during specific time frames in pregnancy. In this regard, it is well-known that after a short period of a pro-inflammatory Th1 immune profile needed for embryo implantation, pregnancy well-being is associated with a shift into a Th2 anti-inflammatory status that dominates until end of pregnancy, when a Th1 pro-inflammatory response is required for parturition (25, 26, 41–49). As IL-33/ST2 axis was shown to be critical in inducing and maintaining a Th2 immune profile both in physiological and pathological settings (35, 50–60), it is possible to speculate that expansion of ST2-expressing B1 B cells is part of the intricate immune mechanism launched during pregnancy to induce and maintain an anti-inflammatory Th2 profile. Supporting this idea, we observed that toward end of pregnancy, when a shift into Th1 profile is demanded, percentages of ST2-expressing B1 B cells were decreased. The decrease observed in the percentages of ST2-expressing B1 B cells between mid- and late pregnancy is somehow in line with previous results from our laboratory, in which we showed that percentages and activation of B1 B cells were decreased during late pregnancy (61). As B1 B cells are the main source of natural antibodies, which are known to be polyreactive and potentially auto-reactive (62), we proposed that decrease of B1 B cells numbers and activation may represent a protective mechanism to prevent the presence of natural and polyreactive antibodies at the fetal–maternal interface, which would put the growing fetus at risk. This is particularly important toward the end of pregnancy when the process of passive immunization takes place. Indeed, it has been shown that IL-33, thought to be ST2, induces B1 B cell activation, leading to a rapid proliferation and production of natural IgM (38). Besides inducing a Th2 immune response, the IL-33/ST2 axis has also been extensively demonstrated to participates in regulating immune homeostasis upon tissue injury (15, 33, 33, 36, 37, 63–69). In the context of pregnancy, Huang and co-authors have recently demonstrated that choriodecidual B1 B cells protect against inflammation-induced PTB by producing progesterone-induced blocking factor 1 (PIBF1) in response to the mucosal alarmin IL-33 (31). Interestingly, we observed that, despite percentages of splenic ST2-expressing B1 B cells dropping toward end of pregnancy, its levels are significantly increased during the acute phase of LPS-induced PTB (5 h after LPS injection). Similarly, we also observed that decidual B cells up-regulated ST2 expression in the acute phase of LPS-induced PTB as compared to term-pregnant mice. This is somehow in contradiction with data from Huang and co-authors who showed that decidual B cells from PTB women express significantly lower levels of ST2 as compared to term-pregnant women (31). This difference can easily be explained by the fact that ST2 expression in decidual B cells was measured in a different time frame. While Huang and co-authors analyzed ST2 expression in B cells of PTB women after delivery, we did this during acute phase of LPS-induced PTB. Indeed, it is widely accepted that in order to maintain tissue homeostasis during inflammatory or infectious insults, white blood cells are recruited to damaged tissues where a transient expression of ST2 is induced and responsiveness to IL-33 is gained (70, 71).

Hence, it can be argued that upon acute inflammation induced by microbial or microbial components such as LPS during pregnancy, decidual B cells rapidly up-regulate ST2 expression acquiring the capacity to respond to IL-33 produced by uterine mucosa and doing so help to prevent or resolve inflammation induced PTB.

Overall, data showed in this work describe for the first time the dynamic of ST2 expression in B cells during normal pregnancy and in preterm birth in mice. This is a remarkable piece of information, and together with a recent publication showing the fundamental role of decidual B cells in preventing inflammation-induced PTB through an IL-33 pathway (31), it places the IL-33/ST2 axis in B cells as a novel and not-yet explored immune mechanisms critical for safeguarding pregnancy well-being. Further studies including human pregnancies are demanded in order to confirm these findings.

## Data Availability Statement

The datasets generated for this study are available on request to the corresponding author.

## Ethics Statement

The animal study was reviewed and approved by the Institutional Commission for the Care and Use of Laboratory Animals (CICUAL), Medical Faculty, Buenos Aires University.

## Author Contributions

NV performed experiments, analyzed data, and wrote the paper. LJ, FQ, and MV performed experiments. DM and MZ contributed with reagents and provided technical support for qPCR displayed in Figure 1. MQ provided technical support for flow cytometry data acquisition. FJ designed experiments, analyzed data, contributed with reagents, supervised the work, and wrote the paper.

### Conflict of Interest

The authors declare that the research was conducted in the absence of any commercial or financial relationships that could be construed as a potential conflict of interest.
