# Supplementary material for: Expression of IL-33 Receptor Is Significantly Up-Regulated in B Cells During Pregnancy and in the Acute Phase of Preterm Birth in Mice
Source: Front Immunol. 2020 Mar 27;11:446. doi: 10.3389/fimmu.2020.00446 (PMC7118206; doi:10.3389/fimmu.2020.00446)
Supplement: Supplementary file 1 [file Data_Sheet_1.pdf]

Supplementary Material

Supplementary Figure 1

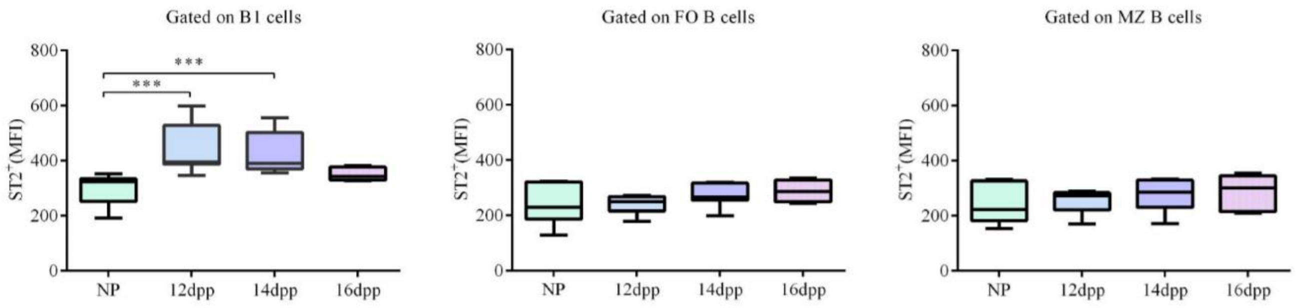

Supplementary Figure 1. Expression levels of ST2 in follicular, marginal zone and B1 B cells in the spleen of pregnant and non-pregnant mice. Box and Whisker plots showing median of fluorescence intensity (MFI) for ST2 in FO, MZ and B1 B cells at day 12 (12 dpp; n=6, n=7 and n=7 respectively), 14 (14dpp; n=10, n=11 and n=11 respectively) and 16 (16 dpp; n=7, n=8 and n=7 respectively) of pregnancy as well as in non-pregnant control females (NP; n=8, n=11 and n=11 respectively). Data are expressed as Box and Whisker plots showing median. ***P<0.001 as analyzed by ANOVA followed by Tukey's multiple comparisons test.
